# Comparative Agronomic Performance and Reaction to Fusarium wilt of *Lens culinaris* × *L. orientalis* and *L. culinaris* × *L. ervoides* derivatives

**DOI:** 10.3389/fpls.2017.01162

**Published:** 2017-07-13

**Authors:** Mohar Singh, Jai C. Rana, Badal Singh, Sandeep Kumar, Deep R. Saxena, Ashok Saxena, Aqeel H. Rizvi, Ashutosh Sarker

**Affiliations:** ^1^National Bureau of Plant Genetic Resources Shimla, India; ^2^National Bureau of Plant Genetic Resources New Delhi, India; ^3^Rafi Ahmad Kidwai, College of Agriculture Sehore, India; ^4^International Centre for Agricultural Research in Dry Areas, South Asia and China Regional Programme New Delhi, India

**Keywords:** lentil, agronomic performance, transgressive segregation, inter-sub-specific, interspecific derivatives, fruitful heterosis, Fusarium wilt

## Abstract

The development of transgressive phenotype in the segregating populations has been speculated to contribute to niche divergence of hybrid lineages, which occurs most frequently at larger genetic distances. Wild *Lens* species are considered to be more resistant against major biotic and abiotic stresses than that of the cultivated species. In the present study, we assessed the comparative agronomic performance of lentil (*Lens culinaris* subsp. *culinaris*) inter-sub-specific (*L. culinaris* subsp. *orientalis*) and interspecific (*L. ervoides*) derivatives, also discussed its probable basis of occurrence. The F_3_, F_4_, and F_5_ inter sub-specific and interspecific populations of ILL8006 × ILWL62 and ILL10829 × ILWL30, respectively revealed a substantial range of variation for majority of agro-morphological traits as reflected by the range, mean and coefficient of variation. A high level of fruitful heterosis was also observed in F_3_ and F_4_ progeny for important traits of interest. Phenotypic coefficient of variation (PCV) was higher in magnitude than genotypic coefficient of variation (GCV) in all generations for several quantitative characters. The results showed high heritability estimates for majority of traits in conjunction with low to high genetic advance in F_3_ and F_4_ generations. Further, F_5_ progeny of ILL10829 × ILWL30, manifested resistant disease reaction for fifteen recombinant inbred lines (RILs) against (*Fusarium oxysporum* f. sp. *lentis* (*Vasd. Srin*.) Gord.). The multilocation agronomic evaluation of both crosses showed better results for earliness, desirable seed yield and Fusarium wilt resistance under two agro-ecological regions of north-western India. These better performing recombinants of ILL8006 × ILWL62 and ILL10829 × ILWL30 can be advanced for further genetic improvement and developing high yielding disease resistant cultivars of lentil.

## Introduction

The Lentil (*Lens culinaris* Medikus subsp. *culinaris*) is a rich in protein (22–35%), minerals (K, P, Fe, Zn) and vitamins for human nutrition (Bhatty, 1988). Globally it ranks sixth position in terms of production among major pulses and the world lentil production constituted 8% of the total dry pulse production (Food and Agriculture Organization, 2015). The important lentil growing countries in the world are India, Canada, Turkey, Bangladesh, Iran, China, Nepal, and Syria (Ahlawat, 2014). The total cultivated area in the world is 4.6 million hectares producing 4.4 million tons of seeds with an average production of 1,095 kg/ha (Food and Agriculture Organization, 2015). Further, wide hybridization as an evolutionary force has a mixed opinion in the literature (Seehausen, 2004) and its possible consequence is the transgressive segregation, which generates novel recombinants that exceed the phenotypic performance of parental lines (Rieseberg et al., 1999). Transgression describes the phenomenon that segregation variance in hybrid population can result in phenotype with extreme character performance exceeding the parental range either in positive or negative direction (Slatkin and Lande, 1994; Rieseberg et al., 1999). One way to enhance genetic variation and potentially increase the performance of commercial cultivars is through incorporation of useful traits of interest found in unadapted gene pool (Tanksley and McCouch, 1997). Because crop wild relatives (CWRs) of most of the domesticated species often carry hidden alleles, which may not be phenotypically obvious, but can be efficiently introgressed into elite genetic backgrounds for improving cultivated species (de Vicente and Tanksley, 1993; Foolad et al., 2002; Singh et al., 2014b). In lentil, modern commercial cultivars have some superiority over traditional varieties in terms of their yield potential and disease resistance. However, a small number of improved landraces have contributed significantly to the development of these lentil cultivars through pure line and mass selection following hybridization between lines adapted to specific environmental conditions. Notwithstanding the number of lentil varieties released, there has been a limited progress in the production and productivity of this important crop over decades in South Asia including India (Erskine et al., 1998). The pedigree analysis of 35 released varieties of lentil in India has been traced back to only 22 ancestors and only top ten donors contributed 30% to the total genetic base of released cultivars (Kumar et al., 2002). This situation could lead to the crop vulnerability due to pest and disease epidemic as well as unpredictable climatic factors. Therefore, there is an immediate need to synthesize new gene pool of lentil for enhancing genetic gain and that necessitates identification and incorporation of target gene sources (agro-morphological and major biotic stresses including Fusarium wilt) available in wild relatives to develop wider adapting resistant populations against prevailing stresses. This will help in the selection of useful recombinants exhibit transgressive performance for several traits of interest (Koseoglu et al., 2017) with following objectives undertaken into the consideration were (1) to assess the extent of variation in F_3_ and F_4_ generations for important quantitative characters (2) to measure the nature and magnitude of useful heterosis and other genetic variability parameters in F_3_ and F_4_ progeny for important characters and (3) to study the comparative agronomic performance of F_5_ derivatives of both wide crosses including resistance against Fusarium wilt.

## Materials and methods

### Genetic materials, population development, and evaluation

The genetic materials consisting of two cultivated lentil varieties, ILL8006 and ILL10829 of *L. culinaris* subsp. *culinaris* were selected and hybridized with two wild species, ILWL62 of *L. culinaris* subsp. *orientalis* and ILWL30 of *L. ervoides*. The hybridization experiments were conducted during 2010-11 and 2011-12 under glass house condition at the National Bureau of Plant Genetic Resources (NBPGR), Pusa, New Delhi, India (28° 35′ N′, 70° 18′ E, 226 m amsl) and summer Himalayan nursery Experimental Farm at CSK, Himachal Pradesh Agricultural University Research Station, Sangla, India (31° 55′ and 32° 20′ N and 77° 00′ and 79° 50′ E, 2,758 m amsl). The F_1_ hybrids were developed manually and the true hybridity of all F_1_ seeds were also confirmed by Inter Simple Sequence Repeats (ISSR) markers (Singh et al., 2013). Further, F_1_ seeds of both crosses were grown in plastic pots under glass house at NBPGR to obtain F_2_ seeds. In F_2_ generation, 136 plants of inter-sub-specific cross-combination of ILL8006 × ILWL62 and 176 plants of interspecific cross of ILL10829 × ILWL30 were maintained and further advanced through single seed descent (SSD) method of breeding. In F_3_ and F_4_ generations, data were recorded on various quantitative traits viz; days to flowering, days to maturity, plant height (cm), number of branches plant^−1^, number of pods plant^−1^, 100-seed weight (g), seed yield plant^−1^ (g), biological yield plant^−1^ (g), and harvest index (%). The experiments on F_3_, F_4_, and F_5_ progenies were conducted in Augmented Block Design (Federer, 1956) in the research farm of NBPGR New Delhi and NBPGR Regional Station Shimla alongwith one ruling standard check variety (Precoz) under subtropical and temperate climate, respectively. In all experiments, seeds were sown in three rows of 3 m length, 30 cm apart and spaced at 10 cm in each row. The soil type was sandy loam at both locations. One pre-sowing irrigation was also given to ensure adequate seed germination. Recommended agronomic practices were also followed for raising the experimental materials. Total two light rains were experienced during the whole cropping period and necessity of additional irrigation was not felt. Regular hoeing and weeding was also carried out to keep the experimental area free from weeds. No fertilizer doses were applied including other agro-chemicals during the cropping period. At the time of harvesting, shriveled seeds were excluded from the seed yield data and only fully developed seeds were included in the data analysis.

### Statistical analysis

The means were adjusted using online software package for augmented block design developed by Rathore et al. (2004). Before undertaking statistical analysis on the basis of adjusted pooled mean values, homogeneity of variance was tested as suggested by Levene (1960). The quantitative characters were further analyzed for various statistical parameters viz. range, mean, coefficient of variation, fruitful heterosis, and principal component analysis (PCA) using the statistical software SYSTAT-12. Phenotypic and genotypic coefficients' of variation (PCV and GCV) for different traits were calculated as PCV = √VP/ mean × 100, GCV = √VG/mean × 100 as per Burton (1952). Heritability (narrow sense) was estimated as *h*^2^ (ns) = √A/VP × 100 as per Lush (1940). Expected genetic advance was calculated as EGA = *k* × VG/VP × √VP as per procedure of Johnson et al. (1955). Here, *k* = 2.06 (standard value assumed at 5% selection intensity); VG is genotypic variance and VP is phenotypic variance. The numerical data were also subjected to biometrical analysis using SAS software (SAS/Stat, 2011). However, fruitful heterosis (H_F_) coined by Koseoglu et al. (2017) were also estimated over better parent (BP) for selecting superior progeny in both F_3_ and F_4_ generations as: H_F_ (%) = [(F_3_ and F_4_-BP)/BP] × 100%, where, BP is the mean value of the better parent of a particular cross.

### Screening against fusarium wilt resistance

All 176 F_5_ interspecific plant populations of cross ILL10829 × ILWL30 were screened against Fusarium wilt (Fusarium oxysporum f. sp. *lentis* (*Vasd. Srin*.) Gord.) reaction in the wilt sick plot at Pulse Research Farm of Rafi Ahmad Kidwai, College of Agriculture, Sehore Madhya Pradesh, India (23° 12′ N, 77° 05 E, 502 m amsl). The wilt sick plot was maintained following methods given by Bayaa and Erskine (1990), Bayaa et al. (1995, 1997), and Eujayl et al. (1998). All F_5_ interspecific derivatives were sown under Complete Randomized Block Design (CRBD) in two replications of 2.0 m row length and 30 cm apart. The plants were spaced in 10 cm of each row. However, resistant (PL639) and susceptible (L-9-12) checks were repeated every after 15 lines of each replication. The sickness of the soil was tested by raising seedlings of susceptible cultivars, which were wilted completely in 30 days after germination. Data were recorded for all the F_5_ plant populations on alternate days after 15 days. The scale was used to score the disease reaction for Fusarium wilt as suggested by Bayaa and Erskine (1990) and Bayaa et al. (1995, 1997) using 1–9 scale: 1 = no symptoms (highly resistant); 3 = yellowing of the basal leaves only (resistant); 5 = yellowing on 50% of the foliage (moderately susceptible); 7 = complete yellowing of the foliage and partial drying (susceptible); 9 = the whole plant is wilted/dry (highly susceptible).Wilt incidence (percentage of dead plants) was recorded during flowering and pod filling stage.

## Results

**Figure 1:**
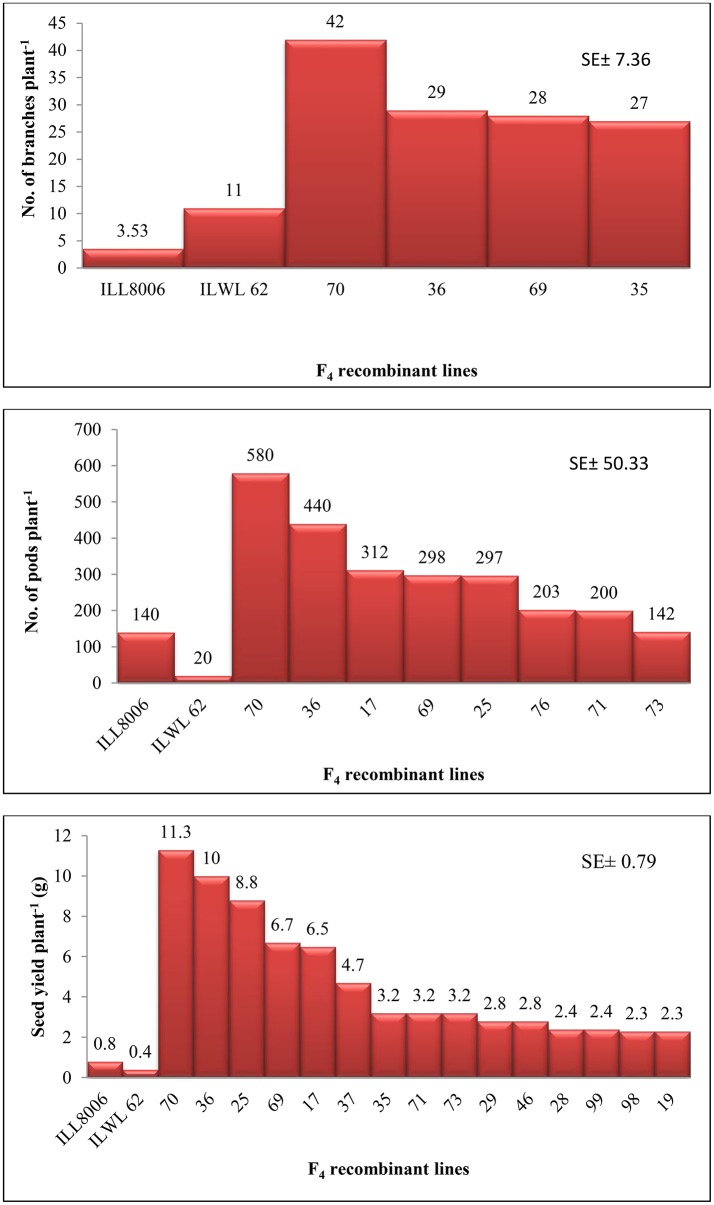
Inter-sub-specific recombinants selected in F_4_ generation of ILL8006 × ILWL62 for number of branches, pods, and seed yield plant^−1^.

**Figure 2:**
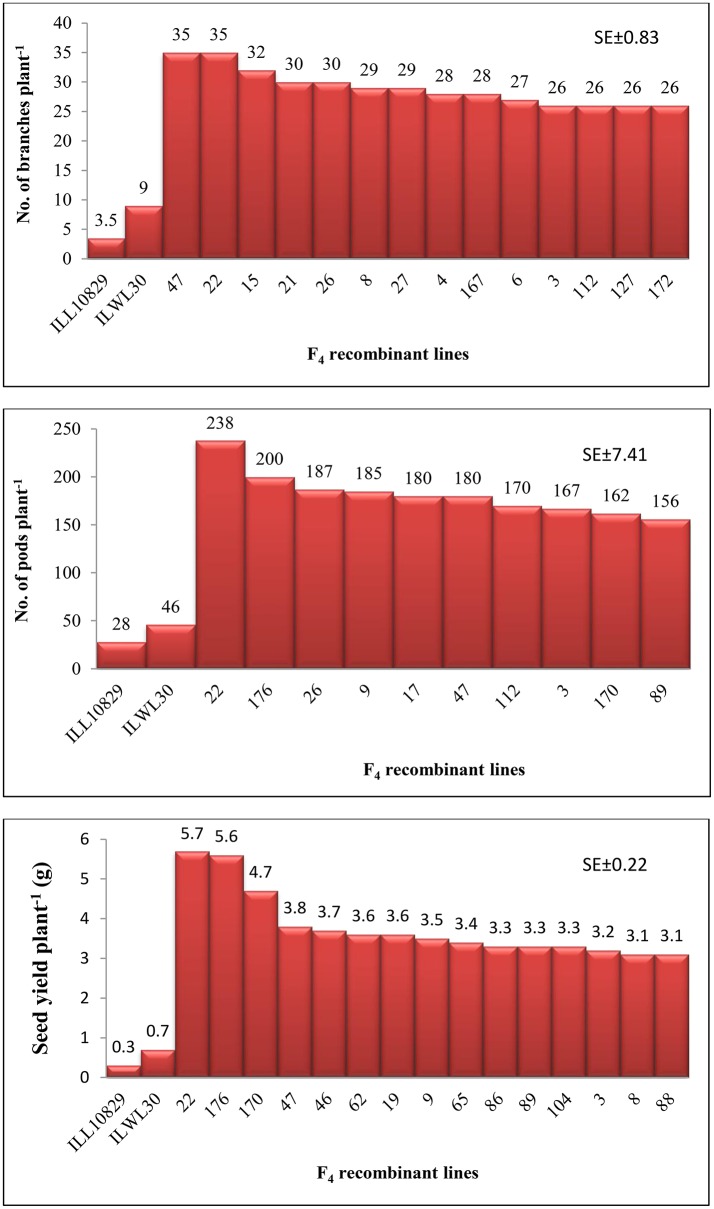
Interspecific recombinants selected in F_4_ generation of ILL10829 × ILWL30 for number of branches, pods, and seed yield plant^−1^.

**Figure 3:**
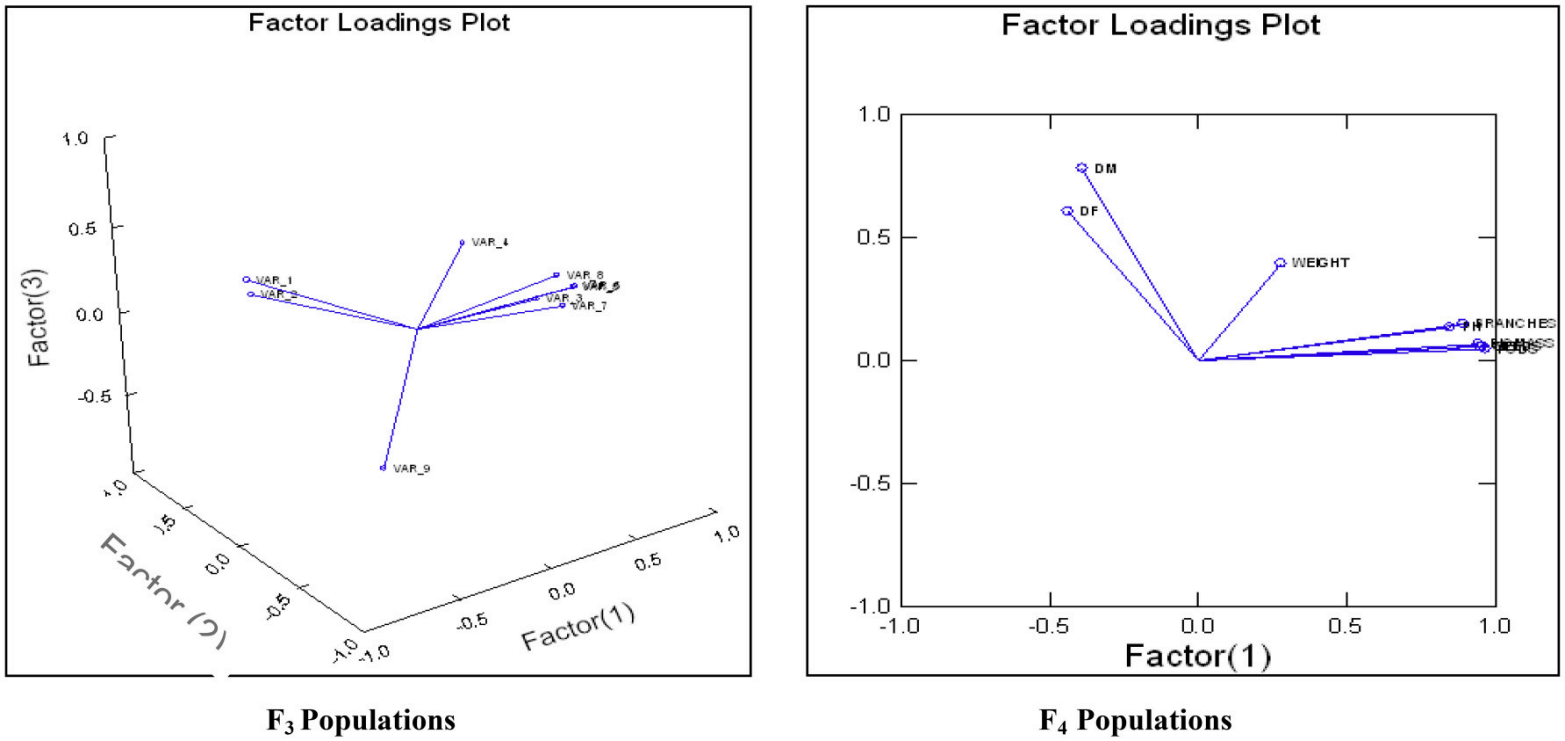
Bi-plot of different variables loaded on principal components in F_3_ and F_4_generation of cross ILL8006 × ILWL62.

**Figure 4:**
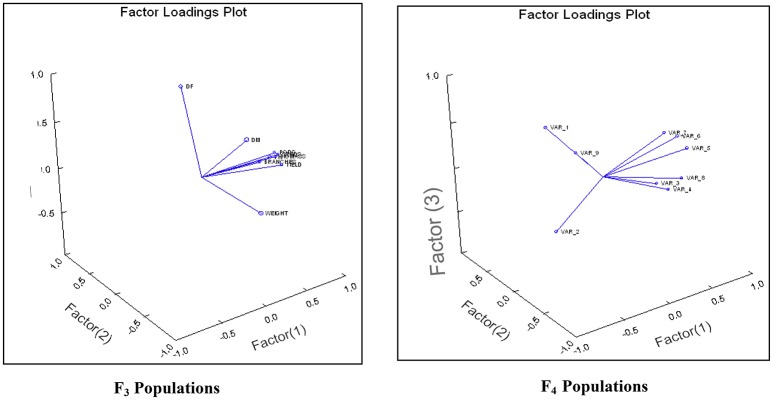
Bi-plot of different variables loaded on principal components in F_3_ and F_4_generation of cross ILL10829 × ILWL30.

**Figure 5:**
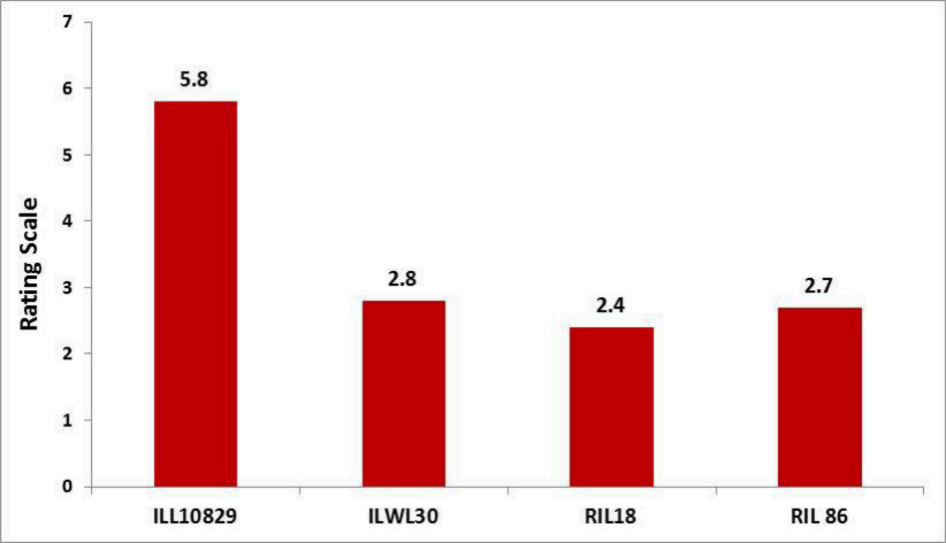
Performance of highly resistant recombinants of cross ILL10829 × ILWL30 and their comparison with parents against Fusarium wilt.

**Figure 6:**
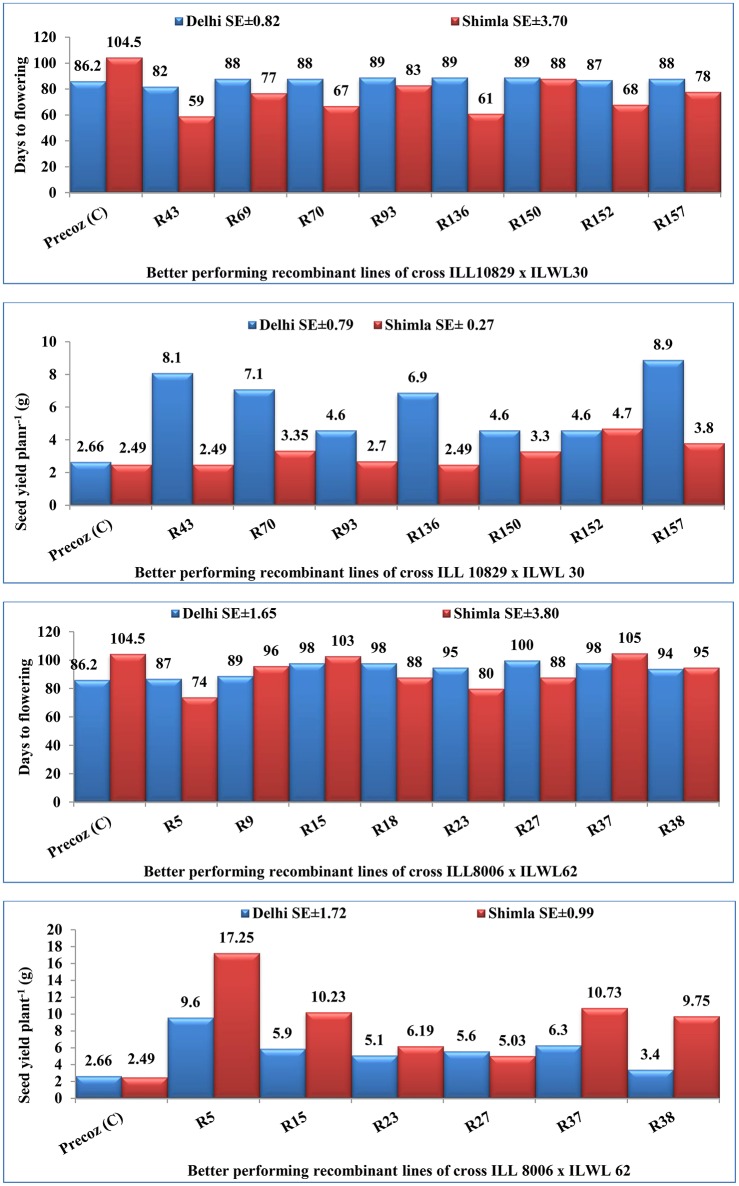
Days to flowering (earliness) and seed yield plant^−1^ of better performing recombinant lines of ILL10829 × ILWL30 and ILL8006 × ILWL62 than check variety (Precoz) based on evaluation tests at two centers.

An attempt was accomplished using cultivated (*L. culinaris* subsp. *culinaris*) varieties taken as female parents (ILL8006 and ILL10829) hybridized with wild species used as male parents, ILWL62 (*L. culinaris* subsp. *orientalis*) and ILWL30 (*L. ervoides*). Overall pod and seed set percentage was calculated as 10.20 and 11.50%, respectively. The results revealed sufficient variability among genetic materials as evident from the analysis of variance (ANOVA) for various traits (significant at *p* = 0.05) studied and it was further reflected by the range, mean and coefficient of variation for majority of characters (Table 1). The range, mean, standard error, and coefficient of variability of F_3_ and F_4_ inter-sub-specific and interspecific derivatives were studied for days to flowering, days to maturity, plant height, number of branches plant^−1^, number of pods plant^−1^, 100-seed weight, seed yield plant^−1^, and biological yield plant^−1^. The mean number of days to flowering and maturity in F_3_ and F_4_ progeny were greater in wild accessions than cultivated female parents. Among two cross-combinations, involving early flowering recipient parents intercrossed with late flowering wild parents. The F_3_ and F_4_ generations were comparable with late flowering and maturing parents. However, large portion of F_3_ and F_4_ progeny of both crosses matured later than the female parents. Likewise, plant height revealed wide range of variation from dwarf to taller plants in all generations of both crosses. There was a substantial variation with respect to number of branches plant^−1^ in F_3_ and F_4_ generations of both crosses. However, for number of pods plant^−1^, large variation was measured from low to high pods and difference in range of F_3_ and F_4_ derivatives was greater than the cultivated parents. Seed yield plant^−1^ also revealed a substantial range of variation from low to high yield in all generations of both crosses and difference in range was observed much higher than the recipient cultivars. Further, the performance of most important characters viz; number of pods plant^−1^ and seed yield plant^−1^, some recombinants produced three to four times greater yield than the cultigen consistently both in F_3_ and F_4_ generations (Figures 1, 2). The nature and magnitude of fruitful heterosis was also assessed in F_2_ derived F_3_ and F_4_ derivatives for days to flowering, days to maturity, plant height, number of branches plant^−1^, number of pods plant^−1^, seed yield plant^−1^, and biological yield plant^−1^ (Table 2). An extent of fruitful heterosis was estimated as percentage of deviation of enhanced progenies from the better parent. In F_3_ generation of cross ILL10829 × ILWL30, heterosis mean performance ranged from −97.33 (seed yield plant^−1^) to 45.77% (number of pods plant^−1^). Likewise, cross-combination of ILL8006 × ILWL62, heterosis mean ranged from −88.64% (Seed yield plant^−1^) to 38.15% (Days to maturity). However, in F_4_ generation of cross ILL10829 × ILWL30, the heterosis mean varied from −98.20% (seed yield plant^−1^) to 39.94% (days to maturity). The cross-combination of ILL8006 × ILWL62, heterosis ranged from −97.80% (seed yield plant^−1^) to 44.65% (days to maturity). Although, there were wide range of variation for majority of traits with respect to heterosis values in F_3_ and F_4_ generations. As far as other genetic parameters are concerned, in general, the extent of phenotypic coefficient of variation (PCV) was higher in magnitude than genotypic coefficient of variation (GCV) for all characters (Table 3). Likewise, heritability along with genetic advance was high for plant height, number of branches plant^−1^, seed yield plant^−1^, and biological yield plant^−1^ in all generations of both crosses. Other characters showed high heritable influence, but genetic advance was low in magnitude. Further, the percent of variation explained by the principal components (PCs) and vector loadings for important agro-morphological traits in different generations are given in Table 4. In F_3_ generation of cross ILL8006 × ILWL62, PC1 accounted for 56.21% of variation, was loaded on plant height, number of branches plant^−1^, number of pods plant^−1^, seed yield plant^−1^ and biological yield plant^−1^, while PC2 accounted for 16.17% of variation, was loaded on 100-seed weight (Figure 3). Whereas, in F_4_ generation of cross ILL8006 × ILWL62, PC1 accounted for 61.83% of variation, was loaded on characters viz; number of branches plant^−1^, number of pods plant^−1^ and biological yield plant^−1^. Likewise, in F_3_ generation of cross ILL10829 × ILWL30, PC1 accounted 54.58% of variation was mainly loaded on 100-seed weight and PC2 accounted for 14.07% of variation and loaded on character days to maturity. While, in F_4_ generation, PC1 accounted for 50.22% variation, was loaded on various traits viz; plant height, number of branches plant^−1^, number of pods plant^−1^, seed yield plant^−1^, and biological yield plant^−1^ (Figure 4). The F_5_ interspecific derivatives of ILL10829 × ILWL30 were screened in the wilt sick plot against Fusarium wilt. The results revealed that all plants of susceptible check variety (L-9-12) were died. There were significant differences among the average percentage of died/wilted plants for 15 recombinant inbred lines (RILs), in which disease incidence was <10%. The mean wilt disease incidence score ranged from 1 to 9 scales with an overall mean of 6.18 and a coefficient of variation of 29.49%. However, the distribution of test entries in incidence against the pathogen showed that only two recombinant inbred lines namely RIL18 and RIL86 performed lowest wilted symptoms (<5%) and were rated as highly resistant (Figure 5) and thirteen other recombinant lines exhibited 6–10% wilted plants, rated as resistant against the pathogen. The wilted plant population severity was recorded from 0 to 90%. An accession ILWC 30 of *L. ervoides* species, exhibited a score of 1–3 with a mean of 2.8 rating. However, recipient parent ILL10829 revealed moderate to susceptible disease reaction with a rating range of 5–7 score. The comparative agronomic evaluation of both crosses revealed a substantial range of variation with respect to important agro-morphological traits (Table 5). Out of 176 recombinant inbred lines (RILs) of cross ILL10829 × ILWL30, seven lines revealed consistent performance both for early flowering and high seed yield under Shimla and Delhi centers (Figure 6). Likewise, in cross ILL8006 × ILWL62, total six recombinant lines manifested desirable performance for early flowering and high seed yield as compared to ruling check variety Precoz in both locations of north-western India.

**Table 1:** Range, mean, standard error, and coefficient of variation for agro-morphological traits in different generations of lentil wide crosses.

**Trait/cross**	**P**_**1**_		**P**_**2**_		**F**_**3**_			**F**_**4**_		
	**Mean ± SE**	**CV (%)**	**Mean ± SE**	**CV (%)**	**Range**	**Mean ± SE**	**CV (%)**	**Range**	**Mean ± SE**	**CV (%)**
**DAYS TO FLOWERING**										
ILL 8006^†^ × ILWL 62	39.5 ± 2.5	8.9	96.0 ± 5.0	7.4	83.0–124	100.0 ± 1.5	12.7	61.0–110	89.2 ± 0.2	15.5
ILL 10829 × ILWL 30	43.0 ± 3.0	9.9	93.5 ± 4.5	6.8	68.0–85.0	80.3 ± 0.2	3.8	58.0–104	65.4 ± 0.9	18.5
**DAYS TO MATURITY**										
ILL 8006 × ILWL 62	72.0 ± 2.0	3.9	122 ± 2.0	2.3	135–150	138.1 ± 0.4	2.2	132.0–152	144.6 ± 0.7	3.8
ILL 10829 × ILWL 30	81.5 ± 2.5	4.3	122 ± 1.5	1.7	132–135	132.9 ± 0.1	0.6	127.0–147	139.9 ± 0.1	1.6
**PLANT HEIGHT (cm)**										
ILL8006 × ILWL 62	19.0 ± 1.0	7.4	26.5 ± 2.5	13.3	5.0–32.0	19.2 ± 0.7	29.6	7.0–34.0	15.4 ± 0.8	38.5
ILL 10829 × ILWL 30	22.0 ± 1.0	6.4	11.0 ± 1.0	12.8	9.0–37.0	22.8 ± 0.3	17.3	6.0–29.0	17.9 ± 0.3	24.2
**NO. OF BRANCHES PLANT^−1^**										
ILL 8006 × ILWL 62	3.2 ± 0.3	14.4	9.7 ± 1.2	18.0	9.0–68.0	35.3 ± 1.7	39.0	3.0–42.0	14.6 ± 1.0	52.5
ILL 10829 × ILWL 30	3.3 ± 0.1	5.0	8.5 ± 0.5	8.2	5.0–54.0	29.9 ± 0.8	33.6	3.0–35.0	14.7 ± 0.5	46.9
**NO. OF PODS PLANT^−1^**										
ILL 8006 × ILWL 62	117.0 ± 23.0	27.8	18.0 ± 2.0	15.7	5.0–589.0	128.1 ± 15.4	110.3	3.0–580.0	104.8 ± 12.6	103.5
ILL 10829 × ILWL 30	26.5 ± 1.5	8.0	39.0 ± 7.0	25.3	7.0–580.0	145.7 ± 8.9	77.2	5.0–238.0	70.3 ± 3.6	64.9
**SEED YIELD PLANT**^−1^ **(g)**										
ILL 8006 × ILWL 62	0.7 ± 0.1	9.3	0.3 ± 0.1	20.0	0.1–5.8	1.4 ± 0.1	108.4	0.1–11.3	2.2 ± 0.3	105.9
ILL 10829 × ILWL 30	0.2 ± 0.1	28.0	0.5 ± 0.2	56.0	0.1–15.4	2.6 ± 0.2	104.1	0.1–5.7	1.8 ± 0.1	59.5
**BIOLOGICAL YIELD PLANT**^−1^**(g)**										
ILL 8006 × ILWL 62	8.3 ± 0.5	9.2	3.0 ± 0.1	4.6	1.0–42.2	12.4 ± 1.0	68.4	2.0–49.0	7.9 ± 1.0	93.9
ILL 10829 × ILWL 30	6.3 ± 2.1	47.5	1.3 ± 0.3	36.3	1.9–39.1	12.0 ± 0.5	55.2	0.7–25.8	8.7 ± 0.4	55.4

† *P_1_ (female parents) ILL8006; ILL10829; P_2_ (male parents) ILWL62; ILWL30; ILWL, international legume wild lentil; ILL, international legume lentil*.

**Table 2:** Estimates of fruitful heterosis (%) in F_2_ derived F_3_ and F_4_ wide cross populations for agro-morphological traits.

**Trait/Cross**	**Generation**			
	**F**_**3**_		**F**_**4**_	
	**Range**	**Mean ± SE**	**Range**	**Mean ± SE**
**DAYS TO FLOWERING**				
ILL10829 × ILWL30	−32.00 to (−15.00)μ	−19.69 ± 0.24	−42.00 to 4.00	−34.53 ± 0.96
ILL8006 × ILWL62	−17.00 to 24.00	0.01 ± 1.55	−39.00 to 10.00	−10.75 ± 1.87
**DAYS TO MATURITY**				
ILL10829 × ILWL30	32.00 to 35.00	32.92 ± 0.07	27.00 to 47.00	39.94 ± 0.25
ILL8006 × ILWL62	35.00 to 50.00	38.15 ± 0.38	32.00 to 52.00	44.65 ± 0.75
**PLANT HEIGHT (cm)**				
ILL10829 × ILWL30	−91.00 to (−63.00)	−77.16 ± 0.31	−94.00 to (−71.00)	−82.09 ± 0.34
ILL8006 × ILWL62	−95.00 to (−68.00)	−80.78 ± 0.70	−93.00 to (−66.00)	−84.55 ± 0.80
**NO. OF BRANCHES PLANT^−1^**				
ILL10829 × ILWL30	−50.00 to (−16.00)	−23.06 ± 0.80	−67.00 to (−44.00)	−22.24 ± 0.55
ILL8006 × ILWL62	−61.00 to (−32.00)	−28.69 ± 0.69	−57.00 to (−28.00)	−21.35 ± 0.44
**NO. OF PODS PLANT^−1^**				
ILL10829 × ILWL30	93.00 to 480.00	45.77 ± 8.94	−297.22 to (−64.22)	−231.87 ± 3.62
ILL8006 × ILWL62	−95.00 to 489.00	28.25 ± 7.14	−97.00 to 480.00	4.80 ± 14.63
**SEED YIELD PLANT**^−1^**(g)**				
ILL10829 × ILWL30	−100 to (−84.60)	−97.33 ± 0.22	−99.90 to (−94.30)	−98.20 ± 0.08
ILL8006 × ILWL62	−100 to (−94.20)	−88.64 ± 0.19	−100 to (−88.70)	−97.80 ± 0.31
**BIOLOGICAL YIELD PLANT**^−1^**(g)**				
ILL10829 × ILWL30	−98.10 to (−60.90)	−87.96 ± 0.53	−99.30 to (−74.20)	−91.28 ± 0.38
ILL8006 × ILWL62	−99.00 to (−57.80)	−87.60 ± 1.04	−98.00 to (−51.00)	−92.03 ± 1.01

*μ negative range performance recorded in parentheses*.

**Table 3:** Range, mean, standard error, phenotypic, and genotypic coefficient of variation, heritability and genetic advance for agro-morphological traits in F_3_ and F_4_ generations of lentil wide crosses.

**Cross/generation**	**F**_**3**_				**F**_**4**_			
**Trait/ parameter**	**PCV^†^**	**GCV**	**Heritability (%)**	**Genetic advance (%)**	**PCV**	**GCV**	**Heritability (%)**	**Genetic advance (%)**
**ILL8006** × **ILWL62**								
Days to flowering	12.68	11.13	84.78	6.43	15.54	13.45	86.49	7.87
Days to maturity	2.26	1.98	82.72	1.97	3.82	3.31	84.51	2.41
Plant height (cm)	29.62	25.10	81.74	51.19	38.56	33.35	82.53	71.60
No. of branches plant^−1^	39.08	34.30	77.78	32.01	52.58	45.49	76.51	88.19
No. of pods plant^−1^	109.38	90.01	72.30	11.20	13.53	89.58	77.36	17.30
Seed yield plant^−1^ (g)	108.34	95.13	87.81	125.01	106.05	91.73	81.50	834.05
Biological yield plant^−1^ (g)	65.59	57.39	82.50	113.67	93.91	81.25	80.51	216.78
**ILL10829** × **ILWL30**								
Days to flowering	3.79	3.49	77.06	4.60	18.51	17.04	86.05	2.94
Days to maturity	0.70	0.60	81.61	1.48	2.23	2.06	82.03	2.02
Plant height (cm)	17.37	17.20	88.99	37.21	24.28	22.60	83.10	52.76
No. of branches plant^−1^	33.65	30.98	82.07	36.74	46.93	43.21	89.07	88.02
No. of pods plant^−1^	77.23	71.17	72.06	11.43	64.91	59.76	82.04	21.72
Seed yield plant^−1^ (g)	104.41	96.14	82.08	725.65	59.52	54.80	77.07	812.48
Biological yield plant^−1^ (g)	55.32	50.93	72.07	117.18	55.50	51.10	82.02	161.97

† *PCV, phenotypic coefficient of variation; GCV, genotypic coefficient of variation*.

Table 4Progeny wise vector loadings and percentage of variation explained by the principal components.

**Table:** 

**Parameter/generation**	**F**_**3**_		**F**_**4**_			
**ILL8006 × ILWL62**	**PC_1_**	**PC_2_**	**PC_3_**	**PC**_**1**_	**PC_2_**
Eigen values	5.06	1.46	1.03	5.57	1.18
Proportion of variance (%)	5.06	1.46	1.03	5.57	1.18
Cumulative variance (%)	56.21	16.17	11.39	61.83	13.16
Characters with greater weighting	3^†^ 4 5 6 7	8	1 2	4 5 7	1 2

**Table:** 

**ILL10829** × **ILWL30**	**PC**_1_	**PC**_2_	**PC**_3_	**PC**_1_	**PC**_2_	**PC**_3_
Eigen values	4.91	1.27	1.08	4.52	1.23	1.02
Proportion of variance (%)	4.91	1.27	1.08	4.52	1.23	1.02
Cumulative variance (%)	54.58	14.07	11.99	50.22	13.67	11.31
Characters ^*Y*^with greater weighting	8	2	1	3 4 5 6 7	2	1 8

† *1, Days to flowering; 2, Days to maturity; 3, Plant height (cm); 4, Number. of branches plant^−1^; 5, Number of pods plant^−1^; 6, Seed yield plant^−1^ (g); 7, Biological yield plant^−1^ (g); 8, 100-seed weight (g)*.

**Table 5:** Comparative agronomic performance of F_5_ lentil inter-sub-specific and interspecific derivatives under two agro-ecological regions.

**Trait/cross**	**Shimla**			**New Delhi**		
	**Range**	**Mean+SE**	**CV (%)**	**Range**	**Mean+SE**	**CV (%)**
**DAYS TO 50 FLOWERING**						
ILL8006 × ILWL62	64.00 to 139.00	96.26 ± 2.75	17.85	87.00 to 102.00	95.45 ± 0.61	4.35
ILL10829 × ILWL30	56.00 to 103.00	70.12 ± 1.01	15.88	78.00 to 89.00	85.38 ± 0.25	3.60
**DAYS TO MATURITY**						
ILL8006 × ILWL62	175.00 to 230.00	203.00 ± 1.74	5.34	115.00 to 124.00	120.62 ± 0.33	1.87
ILL10829 × ILWL30	147.00 to 209.00	196.93 ± 0.81	4.54	104.00 to 120.00	114.46 ± 0.36	3.88
**PLANT HEIGHT (cm)**						
ILL8006 × ILWL62	10.00 to 55.00	28.13 ± 1.70	37.78	11.00 to 32.00	21.43 ± 0.60	19.31
ILL10829 × ILWL30	12.00 to 56.00	30.52 ± 0.79	28.61	10.00 to 30.00	22.99 ± 0.28	15.39
**NO. OF BRANCHES PLANT^−1^**						
ILL8006 × ILWL62	5.00 to 40.00	18.18 ± 1.60	55.06	7.00 to 39.00	26.57 ± 1.05	27.10
ILL10829 × ILWL30	5.00 to 55.00	21.82 ± 0.90	45.15	4.00 to 42.00	24.48 ± 0.60	30.38
**NO. OF PODS PLANT^−1^**						
ILL8006 × ILWL62	4.00 to 693.00	211.72 ± 30.77	90.76	12.00 to 633.00	245.19 ± 19.00	53.14
ILL10829 × ILWL30	2.00 to 250.00	48.91 ± 3.62	81.38	13.00 to 660.00	234.22 ± 9.55	50.78
**NO. OF SEED POD^−1^**						
ILL8006 × ILWL62	0.70 to 2.05	1.23 ± 0.05	25.24	0.56 to 0.95	0.71 ± 0.01	13.72
ILL10829 × ILWL30	0.33 to 2.00	1.10 ± 0.03	32.60	0.42 to 1.25	0.69 ± 0.01	18.13
**NO. OF SEED PLANT^−1^**						
ILL8006 × ILWL62	5.00 to 1060.00	263.15 ± 42.21	100.18	19.00 to 729.00	345.74 ± 27.15	53.84
ILL10829 × ILWL30	1.00 to 262.00	52.16 ± 4.02	84.81	20.00 to 1146.00	349.94 ± 15.33	54.54
**SEED YIELD/PLANT**^−1^ **(g)**						
ILL8006 × ILWL62	0.05 to 18.06	4.15 ± 0.69	103.68	0.20 to 10.80	3.99 ± 0.36	61.70
ILL10829 × ILWL30	0.03 to 4.70	0.97 ± 0.08	91.71	0.20 to 16.70	6.20 ± 0.28	56.37
**100** to **Seed Weight (g)**						
ILL8006 × ILWL62	0.70 to 6.40	1.66 ± 0.14	51.56	0.50 to 1.50	1.09 ± 0.04	22.83
ILL10829 × ILWL30	0.40 to 3.20	1.80 ± 0.04	24.02	0.40 to 3.20	1.71 ± 0.03	24.34
**BIOLOGICAL YIELD PLANT**^−1^ **(g)**						
ILL8006 × ILWL62	6.88 to 100.26	28.47 ± 3.45	75.68	5.40 to 29.60	15.74 ± 0.93	40.36
ILL10829 × ILWL30	1.99 to 78.03	27.59 ± 1.53	61.19	1.20 to 40.70	17.16 ± 0.63	45.94
**HARVEST INDEX (%)**						
ILL8006 × ILWL62	0.50 to 39.87	14.74 ± 1.77	74.81	1.89 to 38.71	24.16 ± 1.31	37.20
ILL10829 × ILWL30	0.12 to 26.93	4.05 ± 0.38	104.54	3.39 to 113.83	35.53 ± 1.00	35.09

## Discussion

The inter-sub-specific and interspecific derivatives offers a nice opportunity for selecting better recombinants carrying useful traits of interest (Lewontin and Birch, 1966). Classical genetic studies have provided a very fairly convincing evidence for the hypotheses that transgression can result from the expression of rare recessive alleles (Rick and Smith, 1953) and or due to complementary gene action (Vega and Frey, 1980). Our results reported here also confirm the occurrence of transgressive performance of lentil derivatives in different filial generations tested under real field conditions of north-western India. Both the wide cross derivatives were advanced and assessed for their agronomic performance and resistance against (Fusarium oxysporum f. sp. lentis (*Vasd. Srin*.) Gord.) as measured by the range, mean, and coefficient of variation including some other important genetic variability parameters in F_3_,F_4_ and F_5_ generations. The results revealed sufficient variability and differences in range of F_3_ and F_4_ generations for days to flowering and maturity of both crosses was due to early and late flowering segregants and the appearance of late flowering and maturity recombinants in all generations, suggesting fixation of late maturing wild alleles in the background of cultivated varieties (Gupta and Sharma, 2007; Singh et al., 2013). However, other important characters like plant height, number of branches plant^−1^ and pods plant^−1^, substantial variation appeared in all generations, which offers a great opportunity selecting desirable lines for practical breeding purposes with certain trait of interest. There was a wide range of variation with respect to seed yield in both crosses and all the available data suggesting the presence of transgression indicating genetic complementarity between *L. culinaris* subsp. *culinaris, L. culinaris* subsp. *orientalis*, and *L. ervoides* taxa. Further, hybrid vigor has opened a useful era in genetic improvement and is now referred to as heterosis breeding. The consistency in the expression of fruitful heterosis in F_2_ derived F_3_ and F_4_ generations might be principally due to the accumulation of favorable additive genes, which are being fixable. Such segregants may be handled as suggested by Redden and Jensen (1974) for selecting desirable recombinant lines for the development of suitable genotypes. It is further confirmed by estimating some other genetic parameters for characters like plant height, number of branches plant^−1^, seed yield plant^−1^, and biological yield plant^−1^, which reveals high heritability along with high genetic advance might be under the control of additive gene effect and selection would be more effective in the early segregating generations. The traits like days to flowering, days to maturity and number of pods plant^−1^, which expressed high heritability and low genetic advance, indicating non-additive gene effect and under such circumstances, selection would be effective in later generations, when non-additive gene effect would have diminished. The estimate of heritability acts as a predictive instrument in exercising reliability of phenotypic value, helping breeders to make a selection for particular trait of interest, when heritability is high. Likewise, genetic advance is a useful indicator of progress, which can be expected as a result of exercising selection on population. Heritability in conjunction with genetic advance is more useful than heritability alone in predicting effects for selecting the best individual genotype, because additive gene effects are likely to be present (Singh et al., 2014a).

Pattern analysis demonstrates that the most important characters with greater weight contributing to total variation are plant height, number of branches plant^−1^, number of pods plant^−1^, seed yield plant^−1^, and 100-seed weight have potential for further use in lentil genetic improvement. This is an indication of the importance of characters contributing in F_3_ and F_4_ generations of both crosses suggesting their role for broadening the genetic base of cultivated gene pool. Our results supported by the finding of Haddad and Muehlbaurer (1991), in which, three lentil wide cross populations advanced from F_2_ to F_4_ generation by SSD and bulk population (BP) breeding methods and were used to compare the relative efficiency of these methods for maintaining genetic variability and desirable selection opportunities. They suggested that SSD method maintained more genetic variability than BP for most of the characters studied. As far as screening of F_5_ generation of cross ILL10829 × ILWL30 against Fusarium wilt is concern, all plant population of susceptible check variety died indicating that the disease incidence was sufficient for effective screening and selection of resistant genetic materials, and inoculums was homogeneously distributed in the wilt sick plot (Nene and Haware, 1980; Bayaa et al., 1997). There was a substantial variation with respect to the incidence of disease ranging from 0 to 90%, suggesting a chance for selecting useful lines resistant to Fusarium wilt. Further, it can also be suggested that the F_5_ interspecific population derived from interspecific hybridization with *L. ervoides* can be a useful gene source for breeding Fusarium wilt resistant cultivars. This can be easily hybridized with other susceptible but productive lentil varieties by intraspecific or intravarietal hybridization programme (Bayaa and Erskine, 1990; Bayaa et al., 1997). Certain transgressive segregants revealing their better agronomic performance as well as resistance against Fusarium wilt, which could be a useful material for developing high yielding wilt resistant lentil cultivars. The incorporation of potential gene sources from *L. ervoides* is highly desirable, because they possess genes for adaptive variation and Fusarium wilt resistance and may further produce other higher yielding derivatives upon hybridization with cultigens, other than we already achieved in the current study. Furthermore, the common better performing F_5_ recombinant lines selected from Delhi and Shimla centers reveals their wider adaptation and potential for developing high yielding cultivars of lentil and these lines can also be utilized as donors for further cross- breeding purposes in lentil genetic improvement (Singh et al., 2013; Tullu et al., 2013). The review of literature pertinent on widening the genetic base of cultivated lentil in relation to transferring resistance from wild *Lens* species against anthracnose (Fiala, 2006; Tullu et al., 2006; Fiala et al., 2009; Vail and Vandenberg, 2011; Vail et al., 2012), ascochyta blight (Tullu et al., 2010), Stemphylium blight (Podder et al., 2013) and yield attributes (Gupta and Sharma, 2006, 2007; Singh et al., 2013) have been successfully attempted. The current research results are in the line of other successes to transferring resistance against Fusarium wilt including yield related traits from the species *L. ervoides* and subspecies *L. culinaris* subsp. *orientalis* is quite relevant for diversification of cultivated gene pool. The following results were concluded as wild *Lens* taxa are invaluable source of useful genes and alleles for important agro-morphological traits and resistant against Fusarium wilt. We also found considerable fruitful heterosis for important characters both in F_3_ and F_4_ wide cross populations including highly resistant derivatives against Fusarium wilt. Further, multilocation evaluation of these crosses manifested consistent desirable expression against earliness and seed yield under two locations, suggesting their wider adaptation. The useful genetic materials are being advanced for further breeding and desirable selection.

## Author contributions

MS: conceived the study and draft research paper. JR, BS, SK, DS, ASax, and AH: Helped in data recording and analysis. ASar: provided wild species from ICARDA and supported this research in India.

### Conflict of interest statement

The authors declare that the research was conducted in the absence of any commercial or financial relationships that could be construed as a potential conflict of interest.

## References

[B1] Ahlawat I. P. S. (2014). Agronomy – Rabi Crops, Lentil. Division of Agronomy, Indian Agricultural Research Institute, Annual Report rabi crops.

[B2] Bayaa B. Erskine W. (1990). A screening technique for resistance to vascular wilt in lentil. Arab. J. Plant Prot. 8, 30–33.

[B3] Bayaa B. Erskine W. Hamdi A. (1995). Evaluation of wilt lentil collection for resistance to vascular wilt. Genet. Res. Crop Evol. 42, 231–235. 10.1007/BF02431257

[B4] Bayaa B. Erskine W. Singh M. (1997). Screening lentil for resistance to Fusarium wilt: methodology and sources of resistance. Euphytica 98, 69–74. 10.1023/A:1003022830846

[B5] Bhatty R. S. (1988). Composition and quality of lentil: a review. Can. Instit. Food Sci. Technol. 21, 144–160. 10.1016/S0315-5463(88)70770-1

[B6] Burton G. W. (1952). Quantitative inheritance in grasses, in Proceedings of the 454 International Grassland Congress, Vol. 1 (State College, PA: Pennsylvania State College), 277–283.

[B7] de Vicente M. C. Tanksley S. D. (1993). QTL analysis of transgressive segregation in an interspecific tomato crosses. Genetics 134, 585–596. 8100788 10.1093/genetics/134.2.585 PMC1205500

[B8] Erskine W. Chandra S. Chaudhary M. Malik I. A. Sarker A. Sharma B. (1998). A. bottleneck in lentil: widening its genetic base in South Asia. Euphytica 101, 207–211. 10.1023/A:1018306723777

[B9] Eujayl I. Erskine W. Bayaa B. Baum M. Pehu E. (1998). Fusarium vascular wilt in lentil: inheritance and identification of DNA markers for resistance. Plant Breed. 117, 497–499. 10.1111/j.1439-0523.1998.tb01982.x

[B10] Federer W. T. (1956). Augmented Block Design, Hawaii. Planters Rec. 55, 191–208.

[B11] Fiala J. V. (2006). Transferring resistance to Collectotrichum Truncatum from Wild Lentil Species to Cultivated Lentil Species (Lens culinaris subsp. culinaris). MSc thesis, University of Saskatchewan, Saskatoon.

[B12] Fiala J. V. Tullu A. Banniza S. Seguin-Swartz G. Vandenberg A. (2009). Interspecies transfer of resistance to anthracnose in lentil (*Lens culinaris* Medik.) Crop Sci. 49, 825–830. 10.2135/cropsci2008.05.0260

[B13] Food and Agriculture Organization (2015). FAOSTAT Statistical database of the United Nations Food and Agriculture Organization, Rime. Available online at: http://faostat.fao.org

[B14] Foolad M. R. Zhang L. P. Khan A. A. Ninoliu D. Lin G. L. (2002). Identification of QTLs for early blight, Alternaria solani resistance in tomato using backcross populations of a *Lycopersicon esculentum* x *L. hirsutum crosses*. Theor. Appl. Genet. 104, 945–958. 10.1007/s00122-002-0870-z 12582599

[B15] Gupta D. Sharma S. K. (2006). Evaluation of wild Lens taxa for agro-morphological traits, fungal diseases and moisture stress in northwestern Indian hills. Genet. Res. Crop Evol. 53, 1233–1241. 10.1007/s10722-005-2932-y

[B16] Gupta D. Sharma S. K. (2007). Widening the gene pool of cultivated lentil through introgression of alien chromatin from wild Lens subspecies. Plant Breed. 126, 58–61. 10.1111/j.1439-0523.2007.01318.x

[B17] Haddad N. I. Muehlbaurer F. J. (1991). Comparison of random bulk population and single seed descent methods for lentil breeding. Euphytica 30, 641–651.

[B18] Johnson H. W. Robinson H. F. Comstock R. E. (1955). Estimates of genetic and 484 environmental variability in Soybean. Agron. J. 47, 314–318. 10.2134/agronj1955.00021962004700070009x

[B19] Koseoglu K. Adak A. Sari D. Sari H. Ceylan F. O. Toker C. (2017). Transgressive segregation for yield criteria in reciprocal interspecific crosses between *C. arietinum* L. and *C. reticulatum* Ladiz. Euphytica 213:116 10.1007/s10681-017-1903-7

[B20] Kumar S. Gupta S. Chandra S. Singh B. B. (2002). How wide is the genetic base of pulse crops, in Pulses in New Perspective, eds Ali M. Singh B. B. Kumar S. Dhar V. (Kanpur: Indian Society of Pulses Research and Development, Indian Institute of Pulses Research), 34–45.

[B21] Levene H. (1960). Robust tests for equality of variances, in Contribution to Probability and Statistics, ed Olkin I. (Palo Alto, CA: Stansford University Press), 278–292.

[B22] Lewontin R. C. Birch L. C. (1966). Hybridization as a source of variation for adaptation to new environments. Evolution 20, 315–336. 10.1111/j.1558-5646.1966.tb03369.x 28562982

[B23] Lush J. L. (1940). Intrusive collection of regression of offspring on dams as a method 501 of estimating heritability of characters. Proc. Am. Soc. Anim. Prod. 33, 293–301.

[B24] Nene Y. L. Haware M. P. (1980). Screening chickpea for resistance to wilt. Plant Dis. 64, 379–380. 10.1094/PD-64-379

[B25] Podder R. Banniza S. Vandenberg A. (2013). Screening of wild and cultivated lentil germplasm for resistance to Stemphylium blight. Plant Genet. Res. 11, 26–35. 10.1017/S1479262112000329

[B26] Rathore A. Prasad R. Gupta V. K. (2004). Computer aided construction and analysis of augmented designs. J. Indian Soc. Agric. Stat. 57, 320–344.

[B27] Redden R. J. Jensen N. F. (1974). Mass selection and mating systems in cereals. Crop Sci. 14, 345–350. 10.2135/cropsci1974.0011183X001400030001x

[B28] Rick C. M. Smith P. G. (1953). Novel variation in tomato species hybrids. Am. Natural. 88, 359–373. 10.1086/281796

[B29] Rieseberg L. H. Archer M. A. Wayne R. K. (1999). Transgressive segregation, adaptation and specification. Heredity 83, 363–372. 10.1038/sj.hdy.6886170 10583537

[B30] SAS/Stat (2011). User's Guide, Version 8.2. Cary, NC: SAS Institute, Inc.

[B31] Seehausen O. (2004). Hybridization and adaptive radiation. Trends Ecol. Evol. 19, 198–207. 10.1016/j.tree.2004.01.003 16701254

[B32] Singh M. Bisht I. S. Kumar S. Dutta M. Bansal K. C. Sarker A.. (2014a). Global wild annual Lens collection: a potential resource for lentil genetic base broadening and yield enhancement. PLoS ONE 9:e107781. 10.1371/journal.pone.0107781 25254552 PMC4177869

[B33] Singh M. Bisht I. S. Dutta M. Kumar K. Kumar S. Bansal K. C. (2014b). Genetic studies on morpho-phenological traits in lentil wide crosses. J. Genet. 93, 561–566. 10.1007/s12041-014-0409-5 25189260

[B34] Singh M. Rana M. K. Kumar K. Bisht I. S. Dutta M. Bansal K. C. (2013). Broadening the genetic base of lentil cultivars through inter-sub-specific and interspecific crosses of Lens taxa. Plant Breed. 132, 667–675. 10.1111/pbr.12089

[B35] Slatkin M. Lande R. (1994). Segregation variance after hybridization of isolated populations. Genet. Res. 64, 51–56. 10.1017/S0016672300032547 7958831

[B36] Tanksley S. D. McCouch S. R. (1997). Seed banks and molecular maps: unlocking genetic potential from the wild. Science 277, 1063–1066. 10.1126/science.277.5329.1063 9262467

[B37] Tullu A. Banniza S. Taran B. Warkentin T. Vandenberg A. (2010). Sources of resistance to Ascochyta blight in wild species of lentil (*Lens culinaris* Medik.). Genet. Res. Crop Evol. 57, 1053–1063. 10.1007/s10722-010-9547-7

[B38] Tullu A. Bett K. Banniza S. Vail S. Vanderberg A. (2013). Widening the genetic base of cultivated lentil through hybridization of *Lens culinaris* “Eston” and *L. ervoides* accession IG72815. Can. J. Plant Sci. 93, 1037–1047. 10.4141/cjps2013-072

[B39] Tullu A. Taran B. Breitkreutz C. Buchwalt L. Banniza S. Warkentin T. D. (2006). A quantitative-trait locus for resistance to Ascochyta blight (Ascochyta lentis) maps close to a gene resistance to Anthracnose (*Colletotrichum trauncatum*) in lentil. Can. J. Plant Pathol. 28, 588–595. 10.1080/07060660609507337

[B40] Vail S. Strelioff J. V. Tullu A. Vandenberg A. (2012). Field evaluation of resistance to *Colletotrichum truncatum* in *Lens culinaris, Lens erovoids* and *Lens erovoids* x *Lens culinaris* derivatives. Field Crop Res. 126, 145–151. 10.1016/j.fcr.2011.10.002

[B41] Vail S. Vandenberg A. (2011). Genetic control of inter-specific derived and juvenile resistance in lentil to *Colletotrichum truncatum*. Crop Sci. 51, 1481–1490. 10.2135/cropsci2010.07.0436

[B42] Vega U. Frey K. J. (1980). Transgressive segregation in inter and intraspecific crosses of barley. Euphytica 29, 585–694. 10.1007/BF00023206

